# Comment on: Incidence of Adverse Psychiatric Events During the Treatment of Inflammatory Bowel Disease With Biologic Therapies: A Systematic Review

**DOI:** 10.1093/crocol/otz051

**Published:** 2019-12-19

**Authors:** Jessica K Salwen-Deremer, Brittany C Speed

**Affiliations:** Department of Psychiatry, Dartmouth-Hitchcock Medical Center, Lebanon, NH; Department of Medicine, Section of Gastroenterology, Dartmouth-Hitchcock Medical Center, Lebanon, NH; Department of Psychiatry, Dartmouth-Hitchcock Medical Center, Lebanon, NH

Biologics are often critical for the treatment of moderate to severe inflammatory bowel disease (IBD), including Crohn’s disease and ulcerative colitis.^1^ However, they are also associated with a number of adverse effects, ranging from infection to malignancy.^2,3^ It is important for prescribing providers to be aware of these adverse effects before starting patients on these drugs and during drug monitoring to ensure the safety of their patients. Although many adverse effects are well described in the literature, less is known about the possible adverse psychiatric events (APEs). Given the increased risk for anxiety and depression that IBD confers,^4^ understanding the possible exacerbation of these conditions is of particular import.

In this issue, Jain et al report on findings from a systematic review of APEs during treatment with biologics. Their primary outcome was the reporting of depression, anxiety, suicidality, and/or psychosis during treatment with a biologic. The authors extensively reviewed the literature, and a search of both relevant databases and Clinicaltrials.gov yielded an initial 8,907 publications and active trials. Of these studies, 16 met inclusion criteria and only 6 were randomized controlled trials (RCTs). Across the 6 RCTs, a total of 2,663 patients received biologic therapy; of these participants, only 15 reports of APEs (5 depression, 6 anxiety, 4 suicidality, and 0 psychosis) were noted, yielding a rate of 0.49% APEs in RCTs.

The authors conclude that there is insufficient evidence that biologic therapy is associated with increased risk of APEs, though they also raise concerns about how APEs are assessed in these clinical trials. The majority of the studies reviewed did not describe their assessment methods for adverse events, and when adverse events were assessed in a systematic way, psychiatric issues were not included. The authors also indicate that none of the trials specify whether psychiatric issues were part of the exclusion criteria, further highlighting the limited assessment. Thus, it is unclear if and what the relationship between biologic therapy and psychiatric events is, as there appears to be pervasive lack of measurement and reporting.

In general, adverse events are defined by clinicaltrials.gov as any unwanted medical occurrence during the individual’s participation in research, whether that occurrence is considered related to the research.^5^ Clinicaltrials.gov also recommends that adverse events are grouped by body/organ systems using the Medical Dictionary for Regulatory Activities (MedDRA), which includes a psychiatric events category. Adverse events are often assessed using brief, unstructured interviews, wherein participants are asked to report on any problems that occurred since the last study visit. Unfortunately, these methods can be unreliable. Clinicians may miss or underestimate the importance of an event, and there is a low level of agreement between clinicians on which events should be documented.^6^ Regarding clinical trials for biologics, when participants are asked to report on symptoms that occurred since the last study assessment, investigators make the assumption that participants have insight into 1) the potential effects of biologics and relevant/reportable symptoms and 2) changes in their physical and psychiatric functioning. In a population that is often accustomed to discomfort via fatigue,^7^ chronic pain,^8^ and gastrointestinal issues, it is likely that participants could underreport or dismiss changes in symptoms.

It is highly relevant for RCTs of IBD treatment to consider psychiatric events, given the elevated prevalence rates of psychiatric disorders, particularly depression and anxiety, in this population relative to the general population.^9–11^ Notably, the increase in depression and anxiety has been shown to occur after, and not prior to, IBD diagnosis,^12^ indicating that psychiatric symptoms may be a sequelae of IBD. Elevated depression and anxiety in the IBD population have been associated with poor health-related quality of life^13^ and functional disability.^14^ In addition, depression in IBD predicts future risk of relapse, surgery, and hospitalization.^15–17^ Thus, psychiatric symptoms, principally depression and anxiety, may affect important disease and treatment-related outcomes.

Consistent with Jain et al’s interpretations of their findings, we believe that given the prevalence of depression and anxiety in IBD, the low rate of APEs suggests not that these events are rare, but instead that APEs are rarely evaluated and reported. Indeed, it seems likely that psychiatric symptoms could develop or worsen for some patients during a trial; for example, it is well established that corticosteroids are associated with increased psychiatric symptoms, including hypomania, mania, depression, and psychosis.^18,19^ In IBD, prior research has demonstrated that the presence of depression or anxiety can negatively affect treatment course,^20^ indicating that it is not only important to evaluate APEs during treatment, but also to evaluate baseline psychiatric symptoms at the start of treatment.

Jain et al’s review highlights a gap in the current understanding of adverse events in RCTs of biologic therapy for IBD. In particular, it is clear that future research would be strengthened by the inclusion of objective, reliable assessment tools both at baseline and consistently over the course of a trial. For example, the Patient Health Questionnaire 4 (PHQ-4) is a validated ultra-brief 4 question self-report assessment of current depression (2 items) and anxiety (2 items) symptoms.^21^ Other commonly used and well-validated measures of depression, which include suicidality assessment, include the Beck Depression Inventory II (BDI-II)^22^ and the Patient Health Questionnaire 9 (PHQ-9)^23^ and well-validated measures of anxiety include the Generalized Anxiety Disorder 7 (GAD-7)^24^ and the Anxiety Sensitivity Index 3 (ASI-3).^25^ In the event that the self-report assessment indicates elevated symptoms, follow-up with a structured diagnostic assessment may be beneficial in determining the presence of specific diagnoses, such as the Diagnostic Interview for Anxiety, Mood, and OCD and Related Neuropsychiatric Disorders (DIAMOND).^26^ Overall, we recommend that future RCTs in this population evaluate psychiatric symptoms at baseline and as potential adverse events during treatment using validated assessment tools. Furthermore, we also recommend that not only study investigators, but also regulatory agencies, be responsible for comprehensive and valid assessment of APEs. If these valid assessments were to be required as part of funded trials, they would likely be assessed more thoroughly across IBD studies in general, thereby increasing our ability to rely on findings that could be affected by psychopathology.

If investigators do decide to assess psychiatric symptoms in a systematic way, it increases the likelihood that participants will report these concerns, necessitating protocols and clinical decision making. In the case that participants do report severe symptoms of psychiatric illnesses, investigators will likely want to be prepared to hospitalize individuals with serious thoughts about harming themselves or others or who are unable to care for themselves due to psychiatric symptoms. Patients with less acute symptoms may benefit from receiving referrals for evidence-based outpatient psychotherapy (e.g., psychologytoday.com; www.findcbt.org) and/or bibliotherapy or free online evidence-based self-help materials (e.g., Center for Clinical Interventions: https://www.cci.health.wa.gov.au/).

## CONCLUSION

Overall, the literature suggests that individuals with IBD are more likely to experience anxiety and depression, and that depression increases the likelihood of a number of IBD-related complications. Despite these clear associations, Jain et al’s review indicates that APEs are not typically measured or reported in clinical trials of biologics. It is not yet established what psychiatric effects biologic therapy may have in patients with IBD; thus, it is necessary for future research to systematically evaluate these potential outcomes. We recommend that investigators assess for anxiety and depression during clinical trials, that regulatory agencies encourage or require these assessments, and that investigators use validated measures of psychopathology to ensure these important data are not missed or dismissed.
